# Disentangling soil microbiome functions by perturbation

**DOI:** 10.1111/1758-2229.12989

**Published:** 2021-07-28

**Authors:** Adam Ossowicki, Jos M. Raaijmakers, Paolina Garbeva

**Affiliations:** ^1^ Department of Microbial Ecology Netherlands Institute of Ecology (NIOO‐KNAW) Droevendaalsesteeg 10 Wageningen PB 6708 Netherlands; ^2^ Soil and Water Research Infrastructure (SoWa) Biology Centre CAS České Budějovice Czech Republic; ^3^ Institute of Biology, Leiden University Leiden Netherlands

## Abstract

Soil biota contribute to diverse soil ecosystem services such as greenhouse gas mitigation, carbon sequestration, pollutant degradation, plant disease suppression and nutrient acquisition for plant growth. Here, we provide detailed insight into different perturbation approaches to disentangle soil microbiome functions and to reveal the underlying mechanisms. By applying perturbation, one can generate compositional and functional shifts of complex microbial communities in a controlled way. Perturbations can reduce microbial diversity, diminish the abundance of specific microbial taxa and thereby disturb the interactions within the microbial consortia and with their eukaryotic hosts. Four different microbiome perturbation approaches, namely selective heat, specific biocides, dilution‐to‐extinction and genome editing are the focus of this mini‐review. We also discuss the potential of perturbation approaches to reveal the tipping point at which specific soil functions are lost and to link this change to key microbial taxa involved in specific microbiome‐associated phenotypes.

## Introduction

Soil is a complex ecosystem with the highest biodiversity known so far. One gram of surface soil may contain 10^9^–10^10^ prokaryotic cells (bacteria, archaea), 10^4^–10^7^ protists, 100–1000 m of fungal hyphae, and 10^8^–10^9^ viruses (Tecon and Or, 2017; Vos *et al*., 2013). The activity of the soil biota contributes to a wide variety of ecosystem services such as regulating greenhouse gas emission, sequestrating carbon, mitigating erosion, degrading pollutants, suppressing plant pathogens and supplying nutrients for plant growth (Bardgett and van der Putten, 2014). A fundamental trait of soil functioning is its structure: the arrangement of aggregates of varying sizes and three‐dimensional networks of water‐ and air‐filled pores (Rillig *et al*., 2017). The availability of resources and aeration can vary strongly between the soil pores and change over time. Hence, the spatiotemporal heterogeneities in soil create many different niches for soil microbes resulting in an enormous diversity even on a small scale (Vos *et al*., 2013). Spatially structured environments are typically more prone to support coexistence of multiple species that consume the same resource because access to the resource and its metabolic by‐products is conditioned by the structure of the environment.

Most soil niches are low in nutrient availability but this changes when plant roots penetrate and grow throughout the soil matrix changing the physical and chemical properties by the release of polymeric substances and nutritious exudates (Song *et al*., 2021). The narrow interface between plant roots and the soil, also referred to as the rhizosphere, is one of the most dynamic interfaces on earth (Jones *et al*., 2009). The soil microbiome is the reservoir from which the rhizosphere is populated by taxonomically diverse bacteria, fungi and protists, giving rise to numerous intra‐ and interkingdom interactions. These interactions can be positive, neutral or negative and may impact plant growth and plant health (Mendes *et al*., 2013; Philippot *et al*., 2013). However, only in recent years scientists have started to realize how important the impact of these microbe‐microbe and microbe‐plant interactions is. Over the past decade, research on soil and plant microbiomes has increased exponentially, revealing detailed but still largely descriptive information about the tremendous taxonomic and functional diversity. To date, however, our fundamental knowledge of the mechanisms that drive microbiome assembly, diversity and functioning remains largely elusive.

Soil microbes can affect their environment by releasing primary and secondary metabolites. In this way, they not only modify their niche but also affect their neighbours in a positive or negative manner (Pande and Kost, 2017; Scherlach and Hertweck, 2018). Soil and plant‐associated bacteria are able to distinguish among their microbial competitors and fine‐tune their survival strategies (Garbeva *et al*., 2011). Several independent studies reported that the production of specialized metabolites by soil microbes is the direct result of the interaction with other microorganisms in their immediate vicinity (Traxler *et al*., 2013; Tyc *et al*., 2014). For example, soil bacteria that at first do not appear to produce antimicrobials can be triggered to produce specific or broad‐spectrum antibiotics when confronted with other bacterial species under conditions of carbon‐limitation (Garbeva and de Boer, 2009). These results exemplify that microbial activity cannot be studied in isolation but needs to consider or mimic the biotic and abiotic conditions of the soil niches where the microbes typically reside.

The Black Queen Hypothesis (BQH), which encompasses the evolution of dependency between organisms, puts into perspective our current understanding of the interactions within soil microbial communities. According to the BQH, some microorganisms called beneficiaries ‘avoid’ having a function in order to optimize their adaptation to the environment. This loss of function is possible because other ‘helper’ microorganisms in their direct vicinity provide for this function, thereby offering a stable environment. Hence, soil bacteria can be metabolically dependent on other microbes in their direct surroundings. This may also explain, at least in part, that a considerable fraction of the soil microbial community is not (yet) culturable. Considering that the majority of soil microbial species cannot be cultured under laboratory conditions, our knowledge of the taxonomic and functional diversity of soil and plant‐associated microbial communities is primarily based on next generation sequencing, in particular taxonomy‐based amplicon (16S, 18S, ITS) analyses and function‐based marker gene or shotgun metagenome analyses (Tracanna *et al*., 2021). Hence, for many microorganisms, physiological, morphological and ecological characterizations are still lacking due to the absence of isolated and culturable representatives.

Microbial interactions within the complex soil system are key components of soil health and fertility. This is often experimentally demonstrated by growing plants in sterile or semi‐sterile conditions without the full diversity of the soil or rhizosphere microbiome: plant growth promotion or plant disease protection are typically lost or compromised in sterile or semi‐sterile soil conditions. Nevertheless, sterilizing is a very ‘crude’ tool providing only a ‘switch on, switch off’ condition of the soil. The outstanding question is how to disentangle the complex network of microbial interactions and to reveal the key players providing a specific microbiome‐associated phenotype (MAPs; (Oyserman *et al*., 2018)). A promising tool to study functions of microbial communities is the use of a selective pressure that perturbs the microbiome in such a way that the complex structure is simplified in a controlled manner. By comparing the functions provided by native and perturbed (simplified) microbiomes, we can narrow down the relative contribution of the microbiome to that function and identify potential candidates contributing to that function. Among many factors that can cause a microbiome perturbation, some approaches will target specific groups of microorganisms or genes, whereas others will perturb the microbiome in an untargeted way.

The development of molecular techniques allows us to perform targeted microbiome alterations towards disrupting specific genes in the microbiome. In this mini‐review, we describe a selection of different microbiome perturbation approaches used in past and present studies to disentangle the microbial consortia and mechanisms underlying specific microbiome‐associated phenotypes.

This mini‐review focuses on three types of controlled microbiome perturbation: heat, biocides and dilution. Other types of perturbations like drought, flooding, alteration of CO_2_ levels, and xenobiotics will not be discussed here. Many of these perturbations are being studied in the context of climate change and have been recently reviewed in other publications (Jansson and Hofmockel, 2020; Naylor *et al*., 2020b; Veach *et al*., 2020; de Vries *et al*., 2020).

## Perturbation as a tool for deciphering soil microbiome functions

**Fig. 1:**
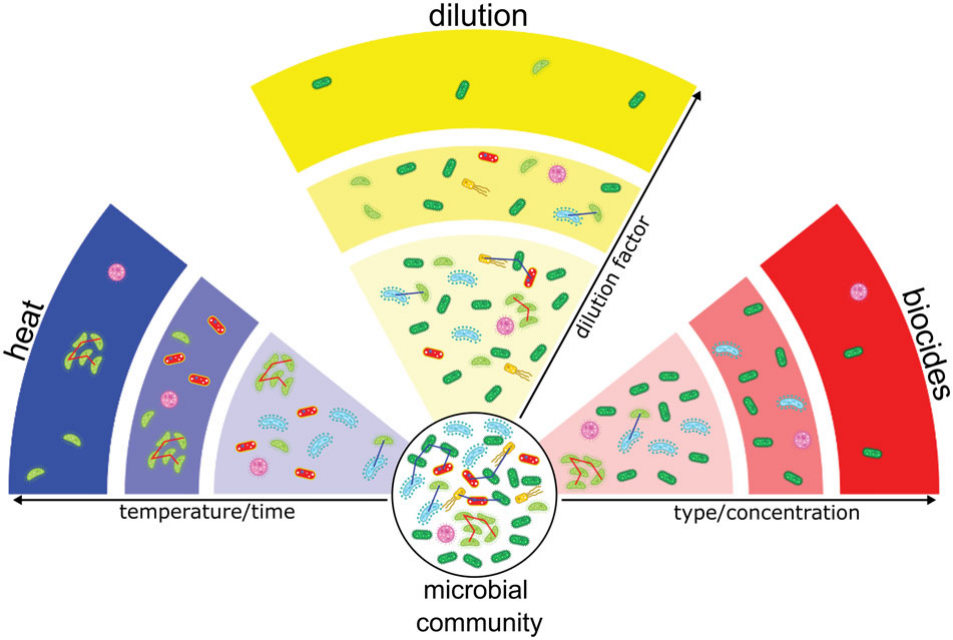
A schematic representation of three perturbation approaches and their effects on a soil microbiome. The original microbial community is presented in the circle and composed of microbial species with different abundances, from highly abundant (dark green), to rare species (pink). The interactions between these microbial species are represented as lines connecting microbes (intraspecific – red; interspecific – blue). The intensity of the perturbation radiates outwards from the inner circle and is represented in three levels with gradually darker colours. Regardless of the perturbation used, the microbial community becomes simplified, the number of species decreases, and the interactions are disturbed. Blue part – heat can be used for sterilization, but when applied at sublethal temperatures and exposure times, it can eliminate only a subset of the original community and not affect a subset with higher resilience to changing temperature and/or able to produce heat resistant survival structures (e.g. spores). Red part – Some groups of microorganisms can be eliminated from the original microbial community by the application of biocides at specific concentrations. That treatment will eliminate those groups of microbes that are susceptible to the compound. We can also attempt to engineer the microbiome using a combination of antibiotics if the presence of resistance genes in a microbial community is known from molecular analyses. Yellow part – The dilution‐to‐extinction approach where the microbial community is distributed stochastically in a series of dilutions. This approach is unspecific and the presence of the groups of microorganisms in consecutive dilutions mostly depends on their initial abundance.

By applying perturbation, we can generate compositional and functional shifts of complex microbial community in a ‘controlled’ way. This microbiome ‘simplification’ reduces the diversity, disrupts the activity of specific members of the microbiome and/or alters the communication and interactions within the microbiome and between microbiome members and other organisms (e.g. insects, plants). Perturbation methods are typically culture‐independent and therefore the preferred choice for soil microbiome analyses where the majority of taxa is yet unculturable. Here, we present an overview of three different microbiome perturbation approaches, namely selective heat, selective biocides and dilution‐to‐extinction (see Fig. 1).

### Perturbation by heat

Already in the 19^th^ century Louis Pasteur discovered that heating wine and milk to a temperature between 60° and 100°C prevents it from spoiling. Pasteur used the heat treatment, later termed pasteurization, to prove the germ theory of disease (Opal, 2009). Elevated temperatures cause substantial mortality in a microbial community and leaves empty niches to be colonized by those microorganisms that survived the heat. For soil microbiomes, the mortality rate depends on the method used to elevate the temperature, the soil moisture level and exposure time. Firmicutes, Actinobacteria and other Gram‐positive genera forming survival structures can typically withstand elevated temperatures. Although much less resistant to heat, also Proteobacteria quickly recolonize heat‐treated soil because of their high growth rates and ability to utilize a broad spectrum of nutritional resources. The community structure in a heat‐treated soil microbiome, measured as diversity and abundance, is unlikely to go back to its initial structure even weeks after the disturbance (Jurburg *et al*., 2017a, 2017b). Riah‐Anglet and colleagues (2015) further investigated the functions of microbial communities after a heat stress by measuring enzymatic activity. Their experiment showed substantial changes in functions and taxa composition. Soil microbiomes did not fully restore specific enzyme activities (β‐glucosidase, cellulase, N‐acetyl‐glucosaminidase, dehydrogenase) within 4 weeks after the heat disturbance. These changes were associated with decreased abundance of Actinobacteria, Acidobacteria and Planctomycetes. They also showed, based on marker‐gene copy number, that soil fungi were more susceptible to the heat stress than bacteria, and that the physical and chemical conditions of the soil have a major impact on the structure of the perturbed community (Riah‐Anglet *et al*., 2015). In this context, Kurm and colleagues (2019) showed that taxa classified as rare (< 0.01% relative abundance) increased their relative abundance even to above 1%. The study by Donhauser and colleagues (2020) addressed changes of bacteria community structure in soils collected from four summits in the Eastern Swiss Alps. The soils were incubated for a month at temperatures ranging from 4° to 35°C. The experiment showed that elevating the temperature above the temperature for optimum growth (set at 27°C–30°C) brings a significant change in community structure caused by increased abundances of fast‐growing taxa, mainly *Burkholderia*‐*Paraburkholderia*, *Phenylobacterium*, *Pseudolabrys*, *Edaphobacter* and *Sphingomonas*. The authors concluded that the upper limit of the optimal growth temperature (30°C) is a tipping point for microorganisms better adapted to higher temperatures and with fast growth rates (Donhauser *et al*., 2020).

Heat perturbation has been widely used to study disease suppressiveness of soils, a microbiome‐associated phenotype that protects plants from root infections despite the presence of a virulent pathogen and a susceptible host plant. Heat perturbation of a soil suppressive to damping‐off disease caused by the fungal root pathogen *Rhizoctonia solani* was investigated in detail by van der Voort and colleagues (2016). They showed that a selective heat treatment of 80°C for 1 h eliminated soil suppressiveness to *R*. *solani* and that the loss of the disease‐suppressive phenotype was accompanied by a decrease in abundance of three Actinobacterial families: Streptomycetaceae, Micrococcaceae and Mycobacteriaceae. If and how these bacterial families contributed to the suppressive phenotype of that soil awaits further validation. Also for other disease‐suppressive soils, the role of specific root‐associated microorganisms has been investigated by heat perturbation. These include the work on *Fusarium*‐suppressive soils (Alabouvette, 1986; Cha *et al*., 2016; Siegel‐Hertz *et al*., 2018), take‐all decline soils (Cook and Rovira, 1976; Duran *et al*., 2017; Raaijmakers and Weller, 1998) and others (for reviews see Gomes Exposito *et al*., 2017; Schlatter *et al*., 2017)).

### Perturbation by biocides

Chemical compounds used to control potentially harmful (micro)organisms in human and animal health as well as agriculture are collectively referred to as biocides and include pesticides (e.g., fungicides, nematicides, insecticides) and antibiotics. The vast majority of studies on biocides concerns the impact of antibiotics on the gut microbiome and human health (for reviews see (Blaser, 2016; Francino, 2016; Schwartz *et al*., 2020)). The effects of biocides on soil and plant microbiomes has been mostly studied in the context of antibiotic and fungicide spills into the environment from anthropogenic sources, like agriculture and industry (Cycoń *et al*., 2019). Nevertheless, the application of biocides can also be a valuable tool for investigating the functionalities of soil microbiomes. Recently, Dong and colleagues (2020) described the impact of different classes of antibiotics on soil bacteriomes and the stability of antibiotics in the soil environment. Their study showed a minor effect of easily degradable antibiotics on soil bacterial communities and a significant growth inhibition of Firmicutes and Bacteroidetes by nalidixic acid and of Firmicutes, Bacteroidetes and Cyanobacteria by tetracycline. These two abovementioned antibiotics also have a negative impact on overall bacterial diversity. Nevertheless, some bacterial groups benefited from the antibiotic treatment. For example, tetracycline, ceftriaxone and nalidixic acid caused an increase in the relative abundance of Betaproteobacteria and Gammaproteobacteria. The authors speculated that this shift might be related to ‘phylogenetic boundaries of antibiotic resistance’ (Dong *et al*., 2020). In recent work, Lee and colleagues (2020) investigated the effect of vancomycin on disease suppressiveness of a soil to bacterial wilt disease caused by *Ralstonia solanacearum*. Their results showed that vancomycin diminished the suppressive phenotype of the soil via disturbing the microbiome composition, in particular the abundance of Firmicutes and Actinobacteria (Lee *et al*., 2020). Similarly, application of fungicides (including those that eliminate oomycetes) can be used to investigate the contribution of soil‐borne fungi to a particular phenotype. For example, Maron and colleagues (2011) elegantly implicated soil‐borne plant pathogens in the plant diversity‐productivity relationship by treating the soil with fungicides (Maron *et al*., 2011). Presumably, the fungicide application had wider implications on microbiome composition and functions but that was not further explored in this study. When using biocides to perturb the soil microbiome, one should also take into consideration biocide stability, dispersion in the system, heat or light sensitivity, and impact of by‐products of metabolism and degradation.

### Perturbation by dilution

Dilution allows perturbation of the microbiome in an untargeted way. Diluted microbial communities contain a subset of the original one and become less diverse and less dense as the dilution increases. Most of the soil microbiome dilution experiments described to date use sterile water or water‐based buffers to extract the microbial community and later as a diluent (Chen *et al*., 2020; Garland and Lehman, 1999; Hol *et al*., 2015; Korenblum *et al*., 2020; Peter *et al*., 2011; Yan *et al*., 2017; Zegeye *et al*., 2019). Following this approach, microbial communities have been serially diluted up to 10^9^ times depending on the study. Microbiome dilutions are mostly introduced back into the sterilized original soil or in a sterile ‘standard’ soil and then incubated for a certain time period before further testing. It is also possible to dilute the soil microbiome without extraction by mixing the non‐sterile soil in different volumes of a sterile background soil.

Studies published to date have shown a reduced taxonomic and functional diversity along the dilution trajectory. For example, Yan and colleagues (2015) compared the taxonomic structure and diversity of the diluted soil microbial community before introduction into soil and 8 weeks after introduction. They showed that dilution leads to a significant change in microbiome assembly over time, but its structure cannot be predicted based on the taxonomic composition of the inoculum (Yan *et al*., 2015). In the follow‐up study, Yan and colleagues (2017) analysed the functional potential of *Jacobaea vulgaris* rhizosphere microbiome over a dilution series using *in‐silico* functional gene prediction based on FOAM database; they showed the strong selective power of the rhizosphere towards microbial functions. In that study the difference between the abundance of the genes assigned to general functions (level 1 in the FOAM annotation) in diluted and undiluted treatments was minimal (Yan *et al*., 2017). Other studies on microbiome dilutions have described changes in various soil/plant traits and functions by sequencing‐independent methods. For example, the carbon source respiration tests showed a non‐linear reduction in the number of the utilized carbon sources in diluted soil microbiomes. Serial dilution of a chitin‐enriched soil microbial community revealed the importance of initial community complexity on the long term stability (Zegeye *et al*., 2019). The impact of diluted soil microbiomes on plant growth and health was extensively studied by Chen and colleagues (2020) who indicated that the loss of bacterial diversity had a negative effect on plant biomass, using lettuce as a model (Chen *et al*., 2020). Another study by Korenblum and colleagues (2020) investigated how the root microbiome modulated the systemic induction of root exudation of metabolites in tomato. They showed that the reduction of microbiome diversity and composition, obtained by dilution‐to‐extinction, resulted in exudation of particular metabolite profiles. The authors associated microbial taxa colonizing roots with the composition of root exudates, especially Bacillales with the increased production of acylsugars esterified with various acyl chains, and Pseudomonadales with secretion of ferulic acid hexose. Hence, by using mixed 'omics techniques, i.e. metatranscriptomics and metabolomics together with 16S taxonomic profiling, they demonstrated that rhizosphere microbiome assembly drives systemically induced root exudation (Korenblum *et al*., 2020).

To study microbiome‐mediated protection of plants from infection, Hol and colleagues (2015) diluted microbial communities from agricultural soils and tested these diluted communities for volatile‐mediated inhibition of plant pathogens. The study revealed that loss of bacterial species resulted in loss of antifungal volatile production. Chemical analysis further revealed that several known antifungal volatiles were only produced in the more diverse and non‐diluted soil bacterial communities (Hol *et al*., 2015). These results led to the hypothesis that bacterial species that produce antifungal volatiles were lost or that specific co‐occurring bacterial species that trigger volatile production in the dominant species were lost. In our recent work, we tested the effect of soil microbiome dilution on disease suppressiveness of soils to *Fusarium culmorum* infections of wheat. Along the dilution trajectory of the soil microbiome, we observed a gradual increase of disease in wheat following a typical sigmoid response curve exemplifying the non‐linear transition from a disease suppressive to a disease conducive soil status (Ossowicki *et al*. unpublished data). 'Omics analyses around the tipping point between the suppressive and conducive soil status is ongoing to obtain more insight into shifts in microbiome composition and functions associated with this microbiome‐associated plant phenotype.

**Fig. 2:**
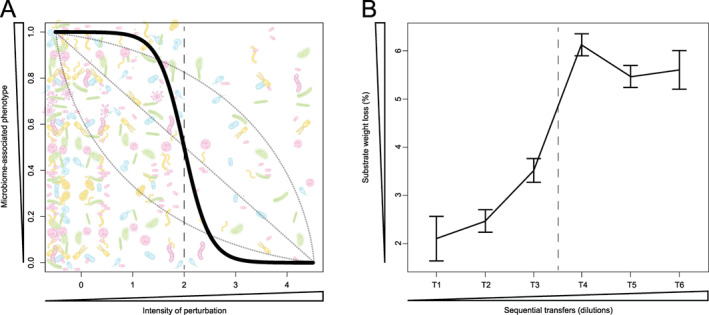
Dynamic changes in microbiome‐associated phenotypes in response to perturbation. A. The black sigmoidal curve represents a hypothetical response of a microbiome‐associated phenotype (e.g. plant growth promotion, disease suppression, greenhouse gas emission) to an increasing intensity of perturbation. With a change in microbiome composition or decline in microbial density due to perturbation, the microbiome‐associated phenotype is little affected at low intensities of perturbation but declines rapidly when a specific threshold intensity of perturbation (often referred to as the ‘tipping point’) is reached. Other types of response (dotted lines) may also be found depending on the mechanistic relationships between the microbiome structure or microbial density and the phenotype involved. An example of reversed situation when a phenotype is gained under stimulation is presented in panel B. B. Example of perturbation leading to an enhancement of a specific microbiome‐associated phenotype. More specifically, the microbiome of Andean forest soil was enriched for microorganisms with lignocellulolytic activity via combined dilutions and media enrichment with lignocellulose rich residues (courtesy Díaz‐García *et al*., 2021; panel B is adapted and modified from the original publication with author permission). In this dilution‐to‐stimulation approach, the microbiome was steered towards a simplified community with enhanced lignocellulolytic activity leading to a sigmoidal increase in substrate degradation (expressed as % weight loss). The dashed line represents the tipping point of the microbiome community change leading to this phenotype.

Last but not least, Díaz‐García and colleagues (2021) recently presented a strategy to assemble a minimal and effective lignocellulolytic microbial consortium by sequential combination of dilution‐to‐stimulation and dilution‐to‐extinction approaches (Díaz‐García *et al*., 2021). After the dilution‐to‐stimulation phase (Fig. 2B), approximately 50 bacterial sequence types were significantly enriched, in particular those belonging to the families Sphingobacteriaceae, Enterobacteriaceae, Pseudomonadaceae and Paenibacillacea. The enriched community secreted an array of enzymes able to degrade xylan, arabinoxylan, carboxymethyl cellulose and wheat straw. The dilution‐to‐extinction method further demonstrated that two selectively enriched bacterial genera (*Pseudomonas*, *Paenibacillus*) were required for effective degradation of the plant polymers. Kang and colleagues (2020) used the same dual approach to select a minimum bacterial consortium able to degrade keratin, allowing them to identify key players in the degradation process (Kang *et al*., 2020).

In conclusion, dilution‐to‐extinction has proven to be a valuable tool to manipulate natural soil microbiomes and to disentangle the role of specific microbiome members in complex microbiome‐driven soil phenomena, such as disease suppressiveness, degradation of complex polysaccharides or root exudation. Unlike other methods, perturbation by dilution allows manipulation of the diversity of a community and to track changes in diversity‐related microbiome characteristics like network structure as well as functional redundancy. Combined with diverse 'omics techniques, dilution‐to‐extinction represents a powerful, yet underused experimental tool to investigate soil microbial functions and microbiome‐plant interactions.

### Other microbiome perturbation approaches

In recent years, we see substantial advances in genome editing tools based on the CRISPR‐Cas system. This method can be used to edit genomes of microorganisms, control gene expression, modulate the production of metabolites and proteins, but also potentially decrease abundance or eliminate members of the microbiome (Ramachandran and Bikard, 2019; Shelake *et al*., 2019). So far, targeted microbiome perturbation using CRISPR‐Cas community editing has been attempted for strain‐specific depletion of members in the mouse gut microbiome. Authors presented a proof‐of‐principal experiment and emphasized the need of highly controlled studies to further develop and refine this approach (Lam *et al*., 2020). In the field of plant‐microbe interactions, this approach was used in the work of Carrión and colleagues (2019), where a specific gene cluster in a member of the plant protective synthetic community was disrupted in a targeted way using the SpyCas9‐mediated system. Disruption of the NRPS/PKS biosynthetic gene cluster led to a partial loss of the disease protective ability of the synthetic bacterial community (Carrión *et al*., 2019). This example highlights that targeted perturbation of a specific function in a soil microbial community presents a powerful tool to reveal complex soil ecosystem services. In addition, a totally different approach to study soil microbiome functions was presented recently by Naylor and colleagues (2020a) where a soil microbiome was enriched in functions by using different nutritional and growth conditions. This work presents an intriguing set of different ‘functional modules’ and their implications for the bacterial community.

## Conclusions

In recent years, soil and plant microbiome research has revealed amazing new insights into microbial diversity, abundance, distribution, dynamics and functions. We have also come to the realization that many microbiome‐associated phenotypes are not due to the action of a single species alone but to multiple interacting species that work together to achieve that function. Microbial network analysis has been frequently used to reveal co‐occurrence among members in the communities and to statistically identify keystone taxa. These taxa can play critical roles in microbial communities and their removal can cause dramatic shifts in microbiome structure and functioning. As ecological microbial interactions are complex, often context‐dependent and include both structural and occasional interactions, it is challenging to disentangle the roles different members of the microbiome have in a particular function or phenotype. Perturbations of different nature (type, magnitude, duration) can help to understand the forces that allow microorganisms to coexist and perform certain functions. Different perturbations allow to design experiments explicitly based on soil ecology and reveal which taxa co‐occur and interact and are responsible for certain soil functions. Furthermore, perturbation experiments are highly instrumental in revealing the tipping point (see Fig. 2) at which a function is lost and to use that for identifying the microbes involved. Furthermore, using an interdisciplinary and integrated approach consisting of state‐of‐the art 'omics techniques and novel bioinformatic approaches in combination with perturbation methods can help reveal the responsible microbes, genes, pathways and metabolites. In conclusion, various perturbation methods have proven to be a valuable tool to manipulate natural soil and plant‐associated microbiomes. As most research in terrestrial microbial ecology to date is focused on bacterial communities, there is yet little knowledge concerning other representatives of soil and rhizosphere microbiome such as fungi, protist, archaea and viruses. Hence, further studies should include these groups to provide a more comprehensive understanding of soil and plant microbiome functioning.

## Conflict of interest

The authors have no conflicts of interest to declare.
